# Smaller and bolder fish enhance ecosystem‐scale primary production around artificial reefs in seagrass beds

**DOI:** 10.1002/eap.3055

**Published:** 2024-11-22

**Authors:** Katrina S. Munsterman, Maximilian H. K. Hesselbarth, Jacob E. Allgeier

**Affiliations:** ^1^ Department of Ecology and Evolutionary Biology University of Michigan Ann Arbor Michigan USA; ^2^ Biodiversity, Ecology, & Conservation Group International Institute for Applied Systems Analysis Laxenburg Austria

**Keywords:** artificial reef, behavior, body size, consumer‐mediated nutrients, individual variation, individual‐based model, production

## Abstract

Effective management of wild animals requires understanding how predation and harvest alter the composition of populations. These top‐down processes can alter consumer body size and behavior and thus should also have consequences for bottom‐up processes because (1) body size is a critical determinant of the amount of nutrients excreted and (2) variation in foraging behavior, which is strongly influenced by predation, can determine the amount and spatial distribution of nutrients. Changes to either are known to affect ecosystem‐scale nutrient dynamics, but the consequences of these dynamics on ecosystem processes are poorly understood. We used an individual‐based model of an artificial reef (AR) and reef fish in a subtropical seagrass bed to test how fish body size can interact with variation in foraging behavior at the population and individual levels to affect seagrass production in a nutrient‐limited system. Seagrass production dynamics can be driven by both belowground (BGPP) and aboveground primary production (AGPP); thus, we quantified ecosystem‐scale production via these different mechanistic pathways. We found that (1) populations of small fish generated greater total primary production (TLPP = BGPP + AGPP) than large fish, (2) fish that foraged more increased TLPP more than those that spent time sheltering on ARs, and (3) small fish that foraged more led to greatest increases in TLPP. The mechanism by which this occurred was primarily through increased BGPP, highlighting the importance of cryptic belowground dynamics in seagrass ecosystems. Populations of extremely bold individuals (i.e., foraged significantly more) slightly increased TLPP but strongly affected the distribution of production, whereby bold individuals increased BGPP, while populations of shy individuals increased AGPP. Taken together, these results provide a link between consumer body size, variation in consumer behavior, and primary production—which, in turn, will support secondary production for fisheries. Our study suggests that human‐induced changes—such as fishing—that alter consumer body size and behavior will fundamentally change ecosystem‐scale production dynamics. Understanding the ecosystem effects of harvest on consumer populations is critical for ecosystem‐based management, including the development of ARs for fisheries.

## INTRODUCTION

Predation is considered to be one of the most important top‐down processes for structuring ecological communities (Estes et al., 2011). In a seminal experiment, Werner et al. (1983) found that the presence of predatory bass influenced the spatial distribution of their prey (sunfish) and that there was a strong interaction between prey body size and foraging behavior, whereby small prey were forced to shelter and forage in habitats with lower quality resources. In many ways, exploitation by humans can similarly affect the structure of ecological communities by reducing the body size (Peres, 2000) and changing the behavior (Ripple & Beschta, 2003) of prey. The implications of these changes for population and community dynamics are well documented, especially in fisheries (Guerra et al., 2020; Januchowski‐Hartley et al., 2011; Robinson et al., 2017). Less considered are the ways in which consumers drive primary production through bottom‐up processes. In many ecosystems, consumer body size and foraging behavior regulate the amount of nutrients supplied (Allgeier et al., 2017; Atkinson et al., 2017; McNaughton et al., 1997; Vanni, 2002), with consequences for primary production, which serves as the basis of energy flow in many ecosystems (DeAngelis et al., 1989; Ryther, 1969). Top‐down processes influence consumer body size and behavior in predictable ways, but what remains less clear is if and how changes in nutrient supply feed back to affect the amount and spatial distribution of primary production in ecosystems.

Consumer body size is the most widely recognized driver of nutrient supply rates because mass‐specific metabolic rates decrease with body size, and thus, smaller individuals excrete more nutrients per unit mass (Allgeier et al., 2015; Brown et al., 2004; Vanni & McIntyre, 2016). Therefore, if predation risk forces small individuals to shelter (McElroy et al., 2018; Werner et al., 1983), one expectation might be that small individuals will have disproportionate effects on primary production near shelter because they (1) spend more time there and (2) supply nutrients at higher rates per unit mass than larger individuals. A less considered attribute in consumer‐mediated nutrients is variation in foraging behavior among individuals within a population. Psychologists and behavioral ecologists have identified a “shy–bold continuum,” whereby shy individuals are often quick to retreat to shelter and bold individuals are more active and exploratory (Wilson et al., 1993). Using acoustic telemetry on snapper species, Allgeier, Cline, et al. (2020) estimated that bold and highly mobile individuals greatly increased (1) the total nutrient supply to mangrove estuaries because movement increased their metabolism and (2) the spatial distribution of nutrients because they had longer foraging bouts. Thus, this study shows that understanding the extent to which individuals differ in their foraging behavior may better predict ecosystem‐scale nutrient cycling than knowing the average effects of individuals within a population. Taken together, understanding how consumer body size and behavior affect nutrient cycling is critical for predicting how changes in consumer populations may affect ecosystem‐scale processes.

Ecosystem‐based management of fisheries considers the implications of fishing on processes beyond those of the target species (Crowder et al., 2008). This concept has long been recognized—Ryther (1969) estimated the primary production required to sustain global fishery production for upper trophic levels and fishery harvest—but is only more recently gaining traction (Chassot et al., 2010; Marshak & Link, 2021). Estimating primary production for fisheries is particularly important in ecosystems with biogenic habitats such as seagrass beds, mangroves, coral reefs, and kelp forests, which themselves serve as the fundamental habitat for fish and their prey. In subtropical seagrass beds that are nutrient limited, artificial reefs (ARs) have been shown to enhance ecosystem‐scale primary production via consumer‐mediated nutrients (Allgeier et al., 2013; Esquivel et al., 2022; Layman et al., 2016). Importantly, the mechanism by which this occurs is that the biogeochemical hotspots created by the concentration of fish excretion causes the seagrass to switch allocation from growth in belowground to aboveground tissues (Allgeier et al., 2013, 2018; Chapin, 1980; Layman et al., 2016). Doing so allows the plant to produce more biomass per unit nutrient than it can in low‐nutrient conditions (e.g., where fish are not aggregating). Given our understanding of these mechanisms, ARs built in seagrass beds are an ideal system to test the implications of changes in fish body size and behavior on belowground (BGPP) and aboveground primary production (AGPP)—the basal resource that fuels secondary production for fisheries.

Here, we used an individual‐based model (IBM) parameterized with empirical data of an AR around which fish shelter to test how fish populations of different body sizes and foraging behaviors affected ecosystem‐scale primary production in a nutrient‐limited seagrass ecosystem. Importantly, to help identify the mechanisms by which the effects occur we asked the following questions for both aboveground and belowground seagrass production dynamics:

How do body size and population‐level foraging behavior independently and interactively mediate primary production?

How does variation in individual‐level foraging behavior interact with body size and population‐level foraging behavior to affect primary production?

## METHODS

### Model environment

To test how population‐level and individual‐level foraging behaviors affect seagrass primary production relative to body size, we used an individual‐based simulation model (arrR R package; Esquivel et al., 2022). The model is parameterized with extensive field data collected in previous studies, which provides fine‐tuned seagrass production parameters and allows us to account for physiological differences associated with fish body size and foraging behavior for fish excretion. Importantly, published model results reflect real‐world patterns from nutrient‐limited systems (Allgeier et al., 2018; Layman et al., 2013). The model simulates movement, foraging, and excretion of fish individuals in a grid‐based seagrass environment. The environment consists of 50 × 50 1‐m^2^ cells, each of which contains water column nutrients, detrital biomass, and belowground and aboveground seagrass biomass. Five cells in the center of the seagrass environment represent an AR on and around which fish individuals can shelter. These cells do not contain belowground and aboveground seagrass biomass but do have nutrients and detrital biomass in the water column. Importantly, the system is closed; that is, nutrients cycle through the system, but no nutrients enter or exit the model environment.

The cycling of nutrients occurs as follows: (1) Water column nutrients are taken up by seagrass and allocated to belowground and aboveground biomass—this allocation changes with nutrient load and subsequently the amount of biomass in these two compartments and is based on empirical data from Layman et al. (2016). (2) A fraction of the standing seagrass (above and belowground) biomass sloughs and directly enters the detrital biomass pool, and a fraction of the detrital biomass is remineralized to the water column nutrient pool. (3) Fish consume nutrients from the detrital biomass pool. (4) Fish recycle nutrients either via excretion that enters the water column nutrient pool directly or through mortality in which case nutrients enter fish detrital biomass, decompose to detrital biomass, and are then remineralized to the water column. (5) Water column nutrients and detritus move through the model environment in a nondirectional manner via diffusion to neighboring cells (see Appendix S1: Figure S1 for modeling scheduling and processes).

#### Seagrass production

Seagrass production is based on a single‐nutrient primary production model following DeAngelis (1992) that allows seagrass to lose biomass as detritus, take up nutrients from the water column, and grow in biomass in belowground and aboveground tissue (Esquivel et al., 2022). Seagrass growth follows basic plant allocation rules in nutrient‐poor systems such that belowground biomass is prioritized over aboveground biomass (Chapin, 1980). Specifically, water column nutrients are allocated first for BGPP to enhance root biomass and then for AGPP once a threshold of standing belowground biomass is met (Esquivel et al., 2022). Nutrient uptake rates, aboveground and belowground biomass values, and allocation thresholds are parameterized with extensive empirical data (Allgeier et al., 2013; Layman et al., 2016; Lee & Dunton, 1999).

To test the effect of consumers on primary production, we quantified BGPP, AGPP, and total primary production (TLPP = BGPP + AGPP; in grams per square meter per day). To understand the spatial distribution of production, we quantified all measures of production in two regions in the seagrass environment: seagrass cells within 5 m of the reef (herein *reef‐adjacent* seagrass production) and those more than 5 m beyond the reef (herein *open* seagrass production). This distinction follows empirical evidence of nutrient enrichment by aggregating fish within a <5m range of ARs built in Caribbean seagrass beds (Allgeier et al., 2013, 2018; Andskog et al., 2023; Brines et al., 2022; Layman et al., 2013).

#### Fish energetics

Fish individuals perform two main functions in the model: (1) consume nutrients from detrital biomass to meet their bioenergetic demands for growth and (2) supply and move nutrients in the environment through excretion. Fish can store excess consumption in individual energy reserves to maintain energetic mass balance when detritus is depleted or when not foraging (see *Fish foraging behavior*). For all fish individuals in the model, we used the energetic parameters for *Haemulon plumierii* (white grunt), a common and commercially important fish species that is common on ARs in the Caribbean (Allgeier et al., 2013; Yeager et al., 2012) and for which there are extensive empirical data (Allgeier, Wenger, & Layman, 2020).

#### Fish foraging behavior

Individual fish behavior includes three different behavior states that are determined largely by their energy reserves and their location in the environment: (1) *Sheltering*: Individuals shelter near an AR cell (<1 m) when their energy reserves are above a threshold of their maximum reserves (e.g., 50%; see *Simulation experiment*). While sheltering, individuals do not forage (Ogden & Ehrlich, 1977; K.S.M. and J.E.A., personal observations, 2023) and solely use their stored energy reserves to maintain energetic mass balance. (2) *Foraging*: When energy reserves fall below the threshold indicated in behavior state 1, individuals move some distance sampled from a log distribution in arbitrary directions to forage on detritus within the open seagrass. (3) *Returning*: Once maximum energy reserves are full, individuals move directly back to the closest AR cell to shelter and do not forage (return to *sheltering*). Importantly, individuals are only in one behavior state each timestep and excrete nutrients throughout all three behavior states, although excretion rates fluctuate with individual activity level (Allgeier, Cline, et al., 2020; Hanson et al., 1997). Behavior states 2 and 3 were combined to calculate the proportion of time that individuals spend in an active movement state (herein *foraging*), while behavior state 1 was used to calculate the proportion of time that individuals spend on the reef (herein *sheltering*).

### Simulation experiment

#### Population‐level and individual‐level foraging behavior

For Question 1 (Q1), population‐level foraging behavior was altered by creating a continuous gradient of the mean foraging time spent by a given population of fish. This was done by varying two energetic parameters: Parameter *A* determines how fast an individual can fill their energy reserves, that is, it sets the maximum detrital biomass that individuals could forage each timestep, and parameter *B* determines how long they can stay sheltering at the AR, that is, it determines the threshold at which an individual can drain their reserves before needing to forage again (Appendix S1: Figure S2). A sensitivity test revealed that changes to parameter *A* had a greater influence on primary production (Appendix S1: Figure S3); thus, parameter *B* was calculated based on a negative curvilinear relationship with parameter *A*. Importantly, in the medium foraging treatment, individuals spent on average around 60% of time foraging and 40% of time sheltering (Appendix S1: Figure S2b), values that are similar to the foraging patterns of white grunts in the wild (Ogden & Ehrlich, 1977).

For Question 2 (Q2), variation in individual‐level foraging behavior was altered by creating populations dominated by bold (forage more), shy (shelter more), and normal (mix of foraging and sheltering behaviors) individuals (Appendix S1: Figure S4). To do this, each individual fish was assigned different values for parameters *A* (by sampling from different distributions) and *B* (by calculating based on the relationship described above). We sampled parameter *A* from (1) a left‐skewed distribution, which resulted in more foraging; (2) a right‐skewed distribution, which resulted in more sheltering; and (3) a normal distribution, which ensured a mix of foraging and sheltering. Altering parameters *A* and *B* for each individual changed the amount of time spent foraging and sheltering, thus creating populations dominated by bold, shy, and normal individuals. Because more active individuals have higher metabolisms (Kerr, 1982) and metabolism rates are positively correlated with excretion rates (Hanson et al., 1997; Schreck et al., 1990), bold individuals that spend more time foraging excrete more nutrients (Allgeier, Cline, et al., 2020).

#### Fish body size

For Q1 and Q2, we created populations of small‐bodied individuals (10 cm) and large‐bodied individuals (18 cm) based on field observations (Munsterman and Allgeier, unpublished). We also used three levels of population biomass (low, medium, and high) that were equal for both small‐ and large‐bodied populations. Population biomass was maintained by setting an initial length and a maximum length for individuals to grow, beyond which individuals die and were replaced with new individuals of the same initial size (Appendix S1: Figure S5). All populations followed the same growth curves, although higher growth rates of small individuals led to slightly increased turnover rates for small‐bodied populations. Importantly, diffusion processes alleviated nutrient pulses from dead fish, resulting in relatively similar detrital pools across small‐ and large‐bodied populations (Appendix S1: Figure S6).

#### Model simulations

While most model processes are deterministic, there are two sources of model stochasticity: (1) stochasticity in model parameters during the initialization (i.e., behavior parameters were sampled from beta and normal distributions to create individual‐level foraging behaviors in Q2) and (2) internal stochasticity of fish movement processes (i.e., movement distances and directions are sampled from lognormal and uniform distributions, respectively, for each timestep in Q1 and Q2). Due to this stochasticity, we simulated all combinations of foraging behavior (population‐level Q1, population‐level and individual‐level Q2), body size, and biomass parameter combinations 50 times (Appendix S1: Table S1). Each combination was simulated for 10 years, and only the final 5 years were used for our analysis to ensure that the ecosystem had reached stable dynamics (Appendix S1: Figure S6).

### Statistical analysis

To test the independent and interactive effects of body size and foraging behavior on seagrass production the following linear models were used:

Q1:Production=body size+population‐level foraging+body size×population‐level foraging

Q2:Production=body size+population‐level foraging+individual‐level foraging+body size×population‐level foraging×individual‐level foraging

We ran separate models for the following response variables: ecosystem‐scale TLPP, AGPP, and BGPP, reef‐adjacent TLPP, and open seagrass TLPP. Because fish biomass is well established to have strong effects (McIntyre et al., 2007), we ran separate models for three biomass levels (low, medium, and high) for each response variable. All continuous variables (production values and Q1 population‐level foraging) were standardized, and square root transformed, and all models met assumptions of normality. Partial eta‐squared values (η_p_^2^) were calculated to assess the proportion of variance explained by each predictor variable using the effectsize package in R (Ben Shachar et al., 2020).

## RESULTS

### Q1: Body size and population‐level foraging on measures of production

**FIGURE 1:**
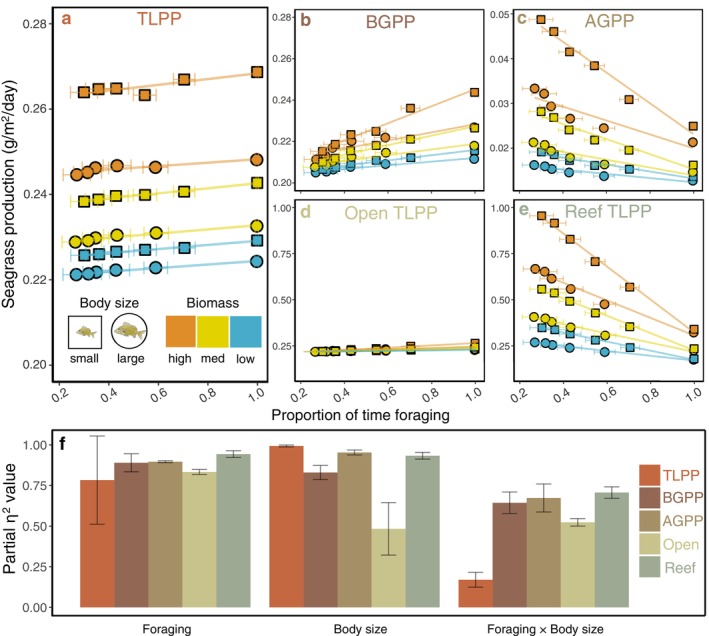
Effects of body size and population‐level foraging on: ecosystem‐scale (a) total primary production (TLPP; belowground primary production [BGPP] + aboveground primary production [AGPP]) in grams per square meter per day, (b) BGPP, (c) AGPP, (d) TLPP in open seagrass (>5 m from the reef), and (e) TLPP adjacent to the reef (<5 m from the reef). Values are means ± SD in production (*y* axis) and proportion of time that individuals in a population spent foraging (*x* axis) across 50 iterations (note the SD for production is small and obscured by the data points). Symbols represent body size (squares for small and circles for large), and colors represent biomass levels (high, medium, and low biomass). Note the different *y* axes due to the spatial scale at which production was calculated: Panels a–c were calculated across the ecosystem‐scale, panels d and e were calculated from cells >5 m from the reef for open TLPP and cells <5 m from the reef for reef TLPP. (f) Partial eta‐squared values (means ± SD across three biomass levels) for independent variables: foraging, body size, and their interaction. Colors correspond to each of the response variables in the five models in panels a–e. Fish illustrations by Katrina S. Munsterman.

Ecosystem‐scale TLPP was positively related to foraging time (greater population‐level foraging; Figure 1a). Across all biomass levels, the effect of foraging (Figure 1f) was large and consistent for BGPP (η_p_^2^ = 0.83–0.9), AGPP (η_p_^2^ = 0.89–0.9), open seagrass TLPP (>5 m from reef; η_p_^2^ = 0.51–0.55), and reef‐adjacent TLPP (<5 m from reef; η_p_^2^ = 0.67–0.74). In comparison, the effect of foraging for TLPP (η_p_^2^ = 0.47–0.95) was more variable than all other measures of production. Body size had a strong effect on all measures of primary production, whereby smaller individuals increased production in all cases.

There was a significant interaction between foraging and body size for both BGPP and AGPP, whereby populations with small individuals that foraged a lot synergistically increased BGPP (Figure 1b) and small individuals that spent most of their time sheltering synergistically increased AGPP (Figure 1c). Notably, behavior and body size trends for BGPP paralleled those of open seagrass TLPP (Figure 1d) and AGPP paralleled those of reef‐adjacent TLPP (Figure 1e). The strength of the interaction between foraging and body size (Figure 1f) was similar for BGPP (η_p_^2^ = 0.58–0.7), AGPP (η_p_^2^ = 0.58–0.75), open seagrass TLPP (η_p_^2^ = 0.51–0.55), and reef‐adjacent TLPP (η_p_^2^ = 0.67–0.74) but much lower for TLPP (η_p_^2^ = 0.12–0.2).

### Q2: Variation in individual‐level foraging on measures of production

**FIGURE 2:**
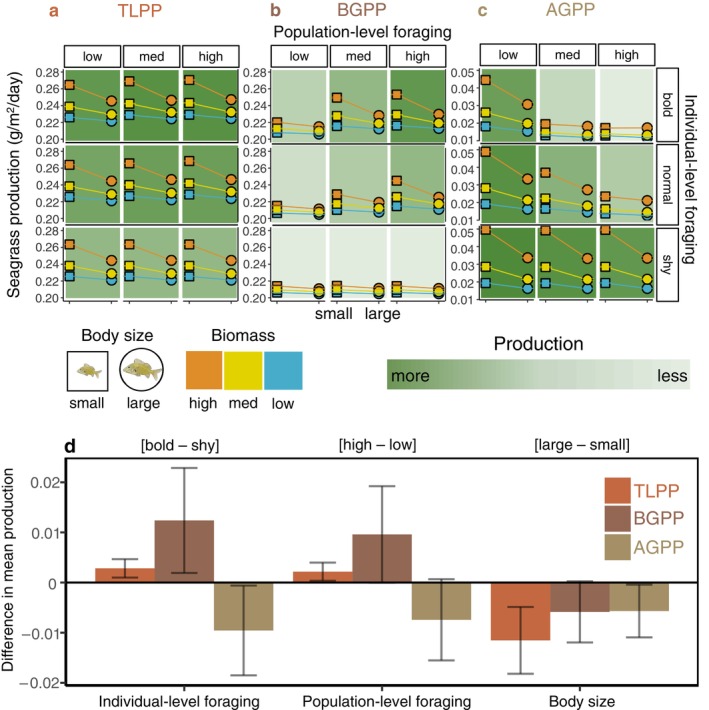
Combined effects of body size, population‐level foraging, and variation in individual‐level foraging on ecosystem‐scale (a) total primary production (TLPP; belowground primary production [BGPP] + aboveground primary production [AGPP]) in grams per square meter per day, (b) BGPP, and (c) AGPP measured in grams per square meter per day (note *y*‐axis scale). Values are means ± SD in production across 50 iterations (note the SD is small and obscured by the data points). Symbols represent body size (squares for small and circles for large), and colors represent biomass levels (low, medium, and high biomass). The background green color gradient illustrates the relative amount of production for each measure, a–c. (d) The difference in mean production ± SD for each independent variable (bold to shy individual‐level foraging, high to low population‐level foraging, large to small body size) was calculated. Colors correspond to each of the response variables in the three models in panels a–c. Fish illustrations by Katrina S. Munsterman.

TLPP was greatest in populations dominated by bold individuals (that foraged more) with high population‐level foraging (high mean foraging time among all individuals in a population) and small body sizes (Figure 2a). BGPP showed trends similar to TLPP (Figure 2b). In contrast, AGPP was greatest in populations dominated by shy individuals (that sheltered more) with low population‐level foraging and small body sizes (Figure 2c).

The positive effect of bold individuals was greatest for BGPP at all three biomass levels (η_p_^2^ = 0.92–0.96; Figure 2d) and less so for TLPP (η_p_^2^ = 0.87–0.96). Bold individuals had a large negative effect on AGPP (η_p_^2^ = 0.95–0.98). Large body sizes had the largest negative effect on TLPP (η_p_^2^ = 0.96–1), followed by BGPP (η_p_^2^ = 0.74–0.97) and AGPP (η_p_^2^ = 0.01–0.58).

## DISCUSSION

Recent efforts have focused on how consumers affect the supply of nutrients to ecosystems, but a critical challenge is quantifying how these inputs affect ecosystem‐scale processes. This is especially important in the context of anthropogenic change because humans are rapidly changing consumer populations. We used a highly parameterized and empirically vetted individual‐based model (IBM) to test how consumer populations of different body sizes and behaviors alter ecosystem‐scale primary production. Because we were able to quantify aboveground and belowground seagrass dynamics, we could identify the specific mechanisms by which these changes occur. We found that populations with smaller individuals had the overall greatest effect on total primary production (TLPP) to the extent that a population of small‐bodied fish can have tantamount effects to populations with greater biomass of large‐bodied fish. Foraging behavior also had substantial effects: TLPP was greatest when the population as a whole foraged more (population‐level behavior). In contrast, high levels of variation in individual‐level behavior had greater effects on where seagrass production occurred: Bold individuals increased belowground production (BGPP) while shy individuals increased aboveground production (AGPP). Given both consumer body size and behavior are heavily impacted by humans, our findings that these factors strongly drive ecosystem primary production are highly relevant for conservation especially if ecosystem‐based management is a priority.

Small and active individuals had the greatest effect on production dynamics, highlighting an overarching outcome of the study: Consumer metabolism governs ecosystem processes. Body size is often a key focus in ecological studies because many ecological processes scale with body size (e.g., excretion: Allgeier et al., 2015; Fritschie & Olden, 2016; Vanni & McIntyre, 2016; and consumption: Munsterman et al., 2021; Ruttenberg et al., 2019). Small individuals with higher rates of metabolism excrete larger quantities of nutrients per unit body mass (Brown et al., 2004). In our study, populations dominated by small individuals drove the highest rates of production across all measures: TLPP, BGPP, and AGPP. Body size effects were most evident in BGPP and were so strong that small individuals at medium biomass (mean size 10 cm at 1.1 kg total biomass) had nearly the same BGPP as populations of large individuals with more than double the biomass (mean size 18 cm at 2.3 kg total biomass). These striking findings portend the strength of the indirect effect humans may have on ecosystem‐scale processes—changing the size structure of populations via overexploitation also has implications for ecosystem primary production.

In addition to body size, individuals that foraged more (as opposed to sheltered) also increased TLPP due to increased metabolism. Population‐level foraging behavior drove different spatial dynamics in seagrass production, particularly when measuring its effects on BGPP and AGPP. Supplemental model runs revealed that across both high and low foraging treatments, BGPP was similar in open and reef‐adjacent seagrass, while AGPP was much greater in reef‐adjacent seagrass where fish sheltered (Appendix S1: Figures S7 and S8). The mechanism by which this occurs in nutrient‐limited systems is that sheltering fish supply nutrients at sufficiently high concentrations that seagrass shifts nutrient allocation from belowground to aboveground tissues near the reef. Field studies from artificial reefs (ARs) built in Caribbean seagrass beds have found enhanced aboveground seagrass production around ARs (Allgeier et al., 2018; Andskog et al., 2023; Layman et al., 2013). These consumer‐mediated nutrient hotspots have been found in other systems as well, including fish on coral reefs (Meyer et al., 1983; Shantz et al., 2015), snow geese in the tundra (Valéry et al., 2010), and river otters at the terrestrial–aquatic interface (Ben‐David et al., 1998). The ecosystem‐scale implications of these localized effects, however, are difficult if not impossible to quantify empirically. Through the use of empirically vetted models, our study quantifies the ecosystem effects of nutrient hotspots and identifies the consumer attributes that determine them. An important yet counterintuitive implication of our study is that ecosystem‐scale primary production may increase as humans promote smaller consumer body sizes and bolder behaviors both directly through harvest and indirectly by removing top predators (Genner et al., 2010; Madin et al., 2016). Andskog et al. (2023) demonstrated empirically that ARs enhance reef‐scale primary production even in heavily fished ecosystems where fish body size is truncated, and our study shows that these impacts extend to the ecosystem scale.

A surprising result from our study was that variation in individual‐level foraging behavior had only a minor positive effect on TLPP despite having substantial effects on where it occurred (BGPP and AGPP). Individual variation has gained a lot of attention in ecology because of the importance of individual‐level traits for ecological processes (Schmitz, 2008; Violle et al., 2012). In nutrient‐poor subtropical systems, individual variation in fish behavior has been shown to alter supply rates and spatial distribution of nutrients (Allgeier, Cline, et al., 2020). In our study, populations dominated by bold individuals who foraged more produced the greatest BGPP because these individuals spent more time in open seagrass and their excreted nutrients were first allocated to belowground tissues, whereas populations dominated by shy individuals who sheltered more produced the greatest AGPP as excess nutrients were allocated to aboveground tissues. Taken as a whole, the net effect was minor but still resulted in an increase in TLPP in populations dominated by bold individuals. This demonstrates that extreme behaviors—either bold or shy—mainly affect where primary production occurs: Bold individuals have greater effects across the seascape for BGPP, while shy individuals are most important for concentrating nutrients near the reef to enhance AGPP. Interestingly, these different behaviors should also change the capacity of seagrass beds to sequester and store carbon. Belowground tissues have been shown to be more important for carbon sequestration (Mazarrasa et al., 2018) and storage (Shayka et al., 2023) than aboveground tissues. Thus, the fact that bold individuals, which are expected to increase with reduced predator populations (Hulthén et al., 2017), should then increase carbon storage in seagrass beds, highlights an additional pathway by which human‐mediated change can alter cryptic belowground dynamics in seagrass ecosystems.

Our study builds on a growing body of evidence that ARs can enhance ecosystem‐scale primary production via nutrients from aggregating fish (Allgeier et al., 2013; Esquivel et al., 2022; Layman et al., 2016). ARs create structures for fish to shelter from predators (Hixon & Carr, 1997), are widely used as a tool for fishery augmentation (Grossman et al., 1997), and are the third most common marine infrastructure globally (Bugnot et al., 2021). However, the utility of AR habitats for fisheries hinges on whether ARs simply attract fish (promoting overfishing) or whether they enhance fish production (Bohnsack, 1989; Powers et al., 2003). Here, we identify the consumer attributes that enhance ecosystem‐scale primary production, which, in turn, should support greater secondary production for fisheries (Layman & Allgeier, 2020; Ryther, 1969). Our findings are partly based on model assumptions that are consistent with established theories and empirical data; future empirical research is essential to provide additional evidence that human‐induced changes to consumer populations may fundamentally alter ecosystems processes. Specifically, our study suggests that shifts in consumer body size to smaller individuals may increase primary production, thus increasing carbon sequestration in seagrass (but see Van Dam et al., 2021). Overfishing of top predators may promote bolder individuals that distribute nutrients across the seascape and enhance belowground tissues for carbon sequestration and storage. Yet, even when consumer populations are heavily influenced by anthropogenetic impacts, we propose that ARs built in biogenic habitats, especially those in nutrient‐limited systems, will enhance primary production and may aid in ecosystem‐based management.

## AUTHOR CONTRIBUTIONS

All authors developed the concepts, questions, and model structure. Katrina S. Munsterman and Maximilian H. K. Hesselbarth coded the model. Katrina S. Munsterman performed statistical analyses. Katrina S. Munsterman wrote the first draft of the manuscript. All authors contributed critically to the drafts and gave final approval for publication.

## CONFLICT OF INTEREST STATEMENT

The authors declare no conflicts of interest.

## Supporting information


Appendix S1.

## Data Availability

Model code (Hesselbarth et al., 2024) is available on Zenodo at https://doi.org/10.5281/zenodo.13859459. Analysis scripts (Munsterman and Hesselbarth 2024) are available on Zenodo at https://doi.org/10.5281/zenodo.12727774.
