# Supplementary material for: Smaller and bolder fish enhance ecosystem‐scale primary production around artificial reefs in seagrass beds
Source: Ecol Appl. 2024 Nov 22;35(1):e3055. doi: 10.1002/eap.3055 (PMC11733711; doi:10.1002/eap.3055)
Supplement: Supplementary file 1 — Appendix S1. [file EAP-35-e3055-s001.pdf]

Smaller and bolder fish enhance ecosystem-scale primary production around artificial reefs in seagrass beds

Ecological Applications

Appendix S1

Katrina S. Munsterman, Maximilian H.K. Hesselbarth, and Jacob E. Allgeier

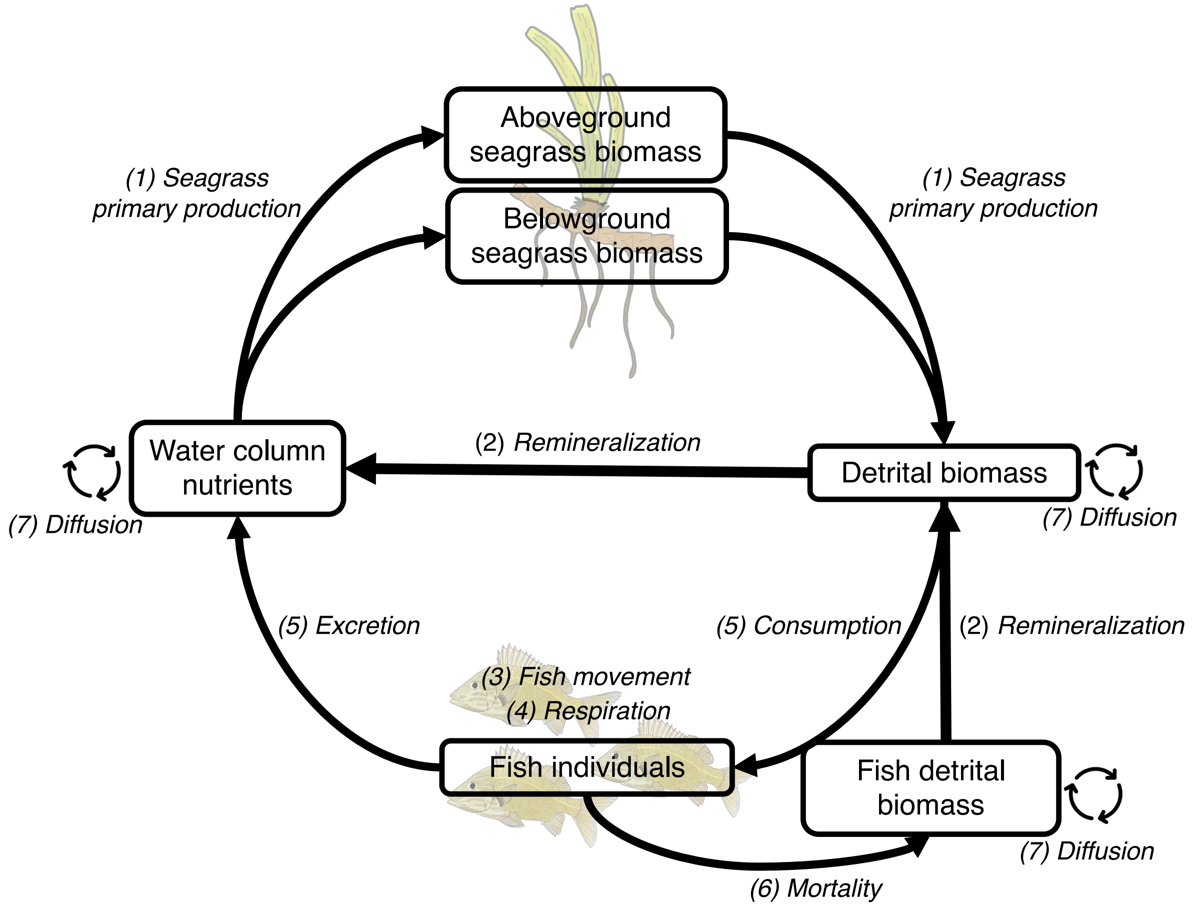

Figure S1. Overview of model subprocesses and scheduling adapted from Esquivel et al. 2022. (*1*) Seagrass primary production is calculated based on available water column nutrients and allocated to aboveground and belowground biomass. A fraction of seagrass biomass is sloughed to detrital biomass. (*2*) Seagrass slough is remineralized to water column nutrients, and dead fish biomass decomposes to detrital biomass to then be remineralized to the water column nutrient pool. (*3*) Fish individuals move around the environment based on behavior state rules. (*4*) Respiration for fish individuals is calculated to determine the amount of (*5*) consumption of detrital biomass needed to meet energetic demands for growth. Excess energy is added to energy reserves. Fish excrete nutrients in their current cells. (*6*) Mortality is calculated for fish that cannot meet energetic demands and fish that reach their maximize size; all dead fish are added to fish detrital pool in their grid cell. (*7*) Nutrients and detritus diffuse across neighboring grid cells. Seagrass and fish illustrations by Katrina S. Munsterman.

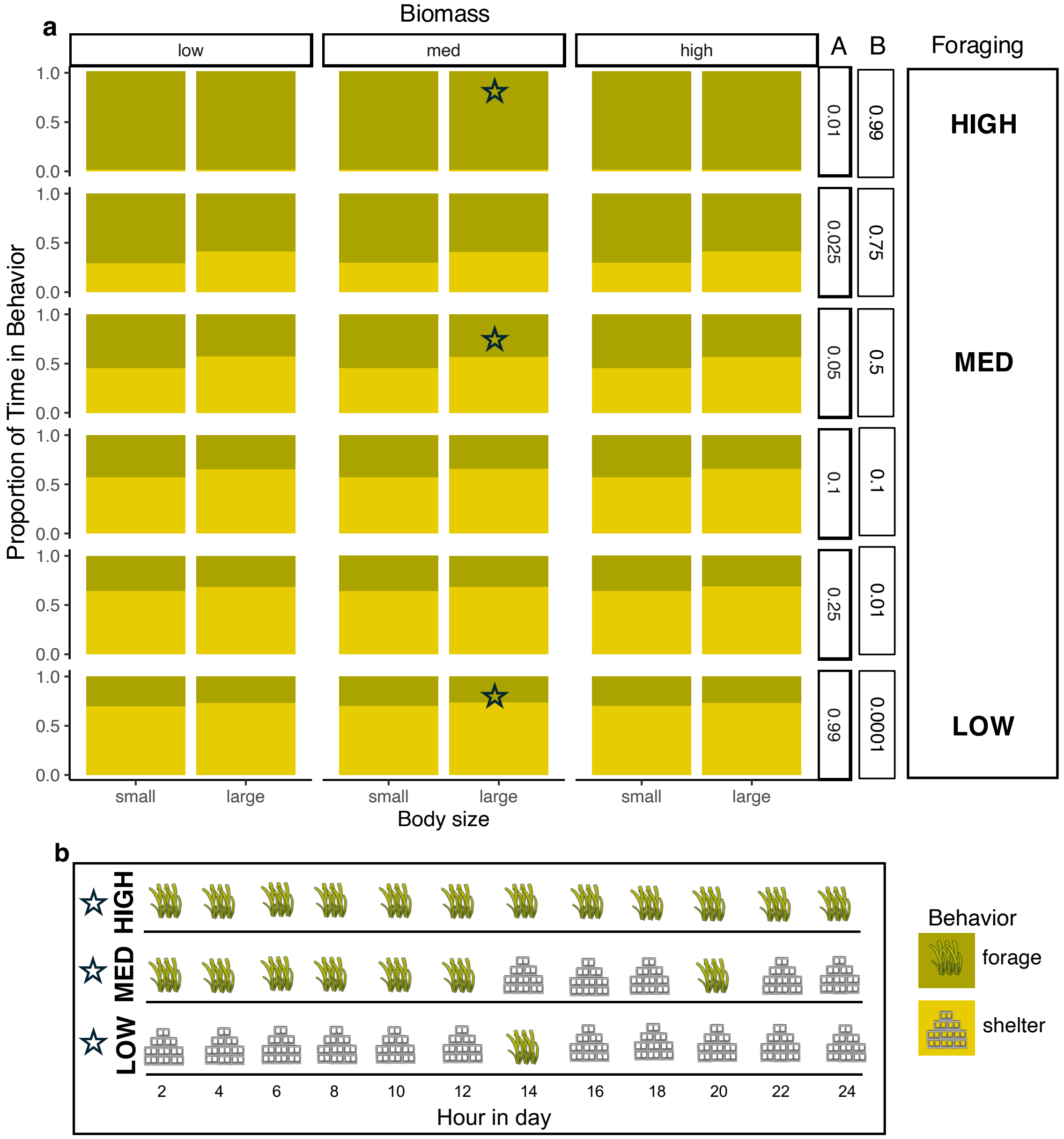

Figure S2. Population-level foraging behavior: (a) Average proportion of time individuals in a population spent either foraging or sheltering based on model Parameter *A*: changes the amount an individual can consume per timestep and Parameter *B*: changes the threshold at which an individual can drain their reserves before needing to forage again. Proportion of time in each behavior varies slightly across body size and biomass treatments. High, medium, and low (indicated on the right side) refer to the population-level foraging behaviors. Three stars refer to (b) an example model run to display the behavior for every 2 hour timestep over a 24-hour period for a large-bodied individual at medium population biomass. Seagrass and artificial reef illustrations: Katrina S. Munsterman.

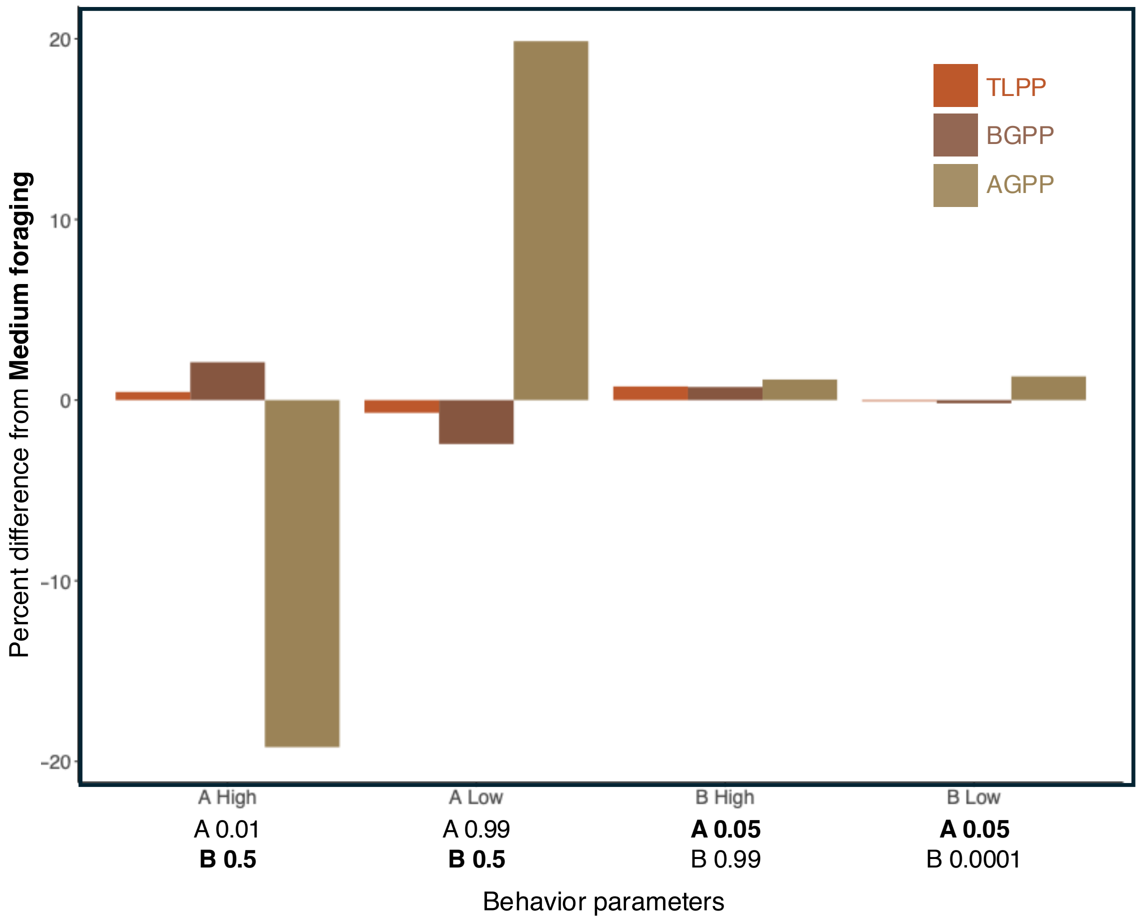

Figure S3. Sensitivity test of population-level foraging behavior parameters. High and Low foraging values from Parameters *A* and *B* were individually altered from Medium foraging (in bold type). Each altered behavior parameter combination (*A* High/*B* Medium, *A* Low/*B* Medium, *A* Medium/*B* High, *A* Medium/*B* Low) and Medium foraging parameter combination (*A* Medium/*B* Medium) was run 25 times for large fish body size and medium population biomass. Percent differences of altered parameter combinations to Medium foraging were calculated from model outputs of total primary production (TLPP), belowground primary production (BGPP), and aboveground primary production (AGPP). Positive values indicate that altered parameter combinations produced increased primary production, while negative values indicate that altered parameter combinations decreased primary production.

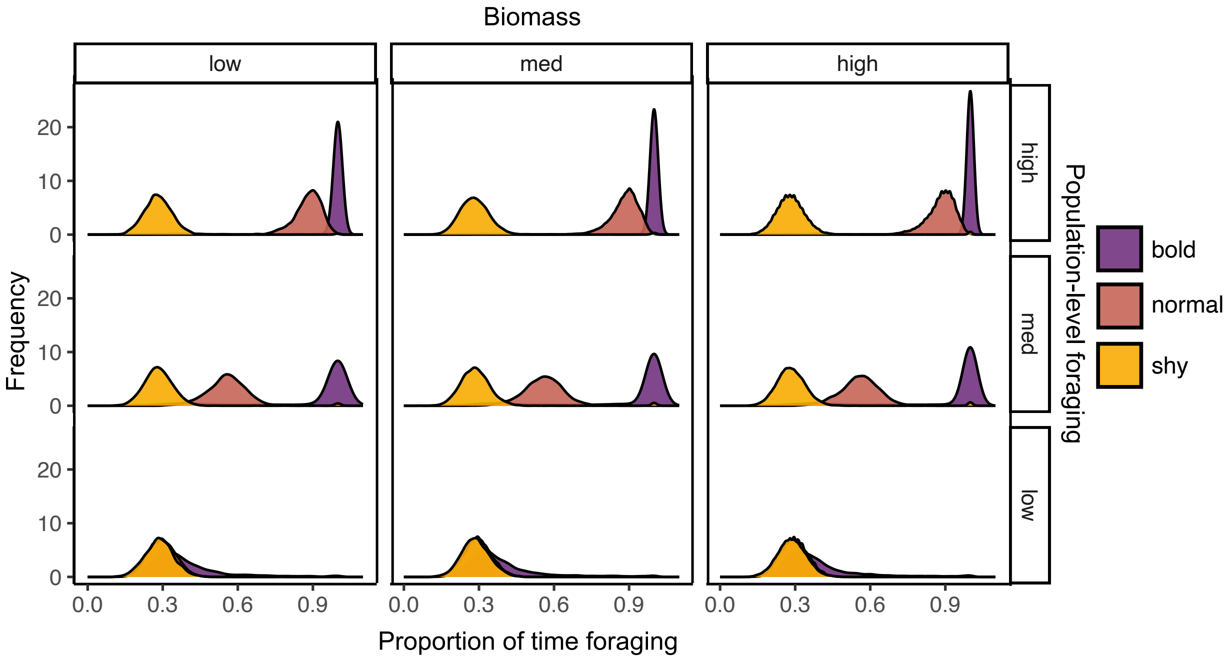

Figure S4. Density plots of individual-level foraging (mean proportion of time foraging for each individual) across three biomass levels and three population-level foraging treatments (mean proportion of time spent foraging across individuals in a population). Data was pooled across small- and large-bodied populations to generate populations dominated by bold individuals (more foraging), shy individuals (more sheltering), and normal individuals (a mix of foraging and sheltering) distributed around the population-level mean.

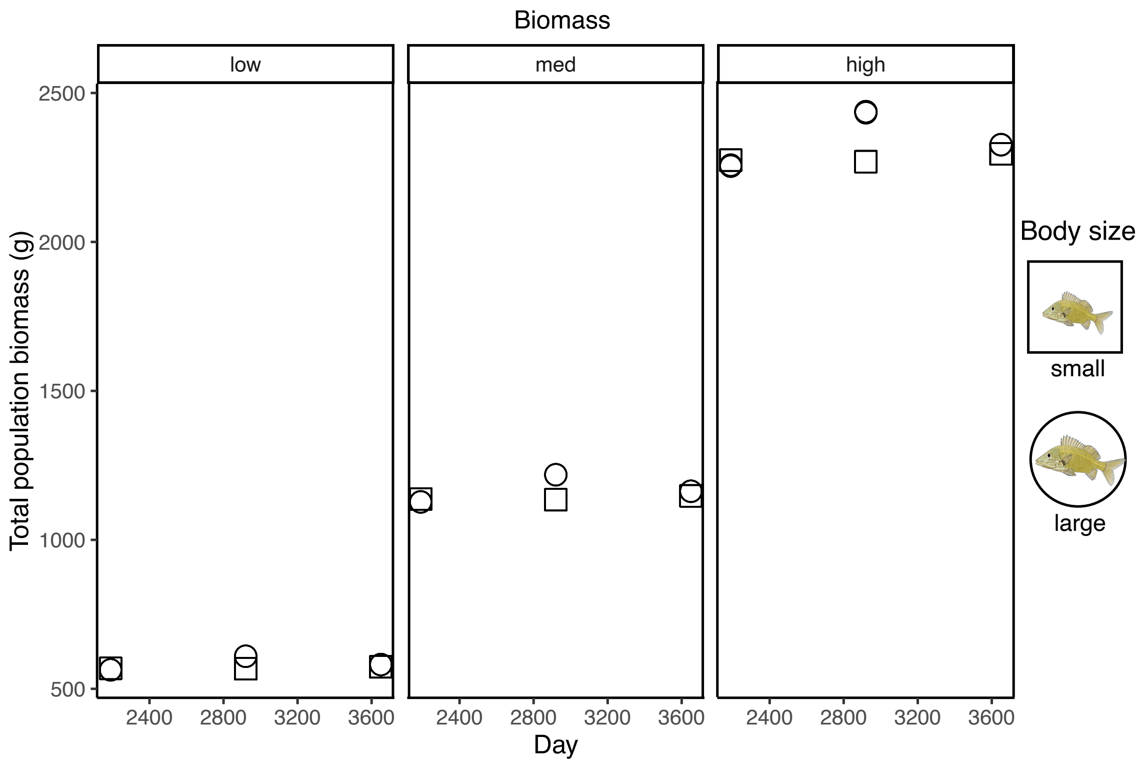

Figure S5. Total population biomass (g) of fish for small (10 cm) and large (18 cm) body sizes at three biomass levels (low, medium, high) from Year 5-10 of simulations (in days). Population biomass was maintained for small- and large-bodied populations by setting an initial length and a maximum length for individuals to grow, beyond which individuals die, were added to a dead fish detrital pool, and were replaced with new individuals of the same initial size. Fish illustrations: Katrina S. Munsterman.

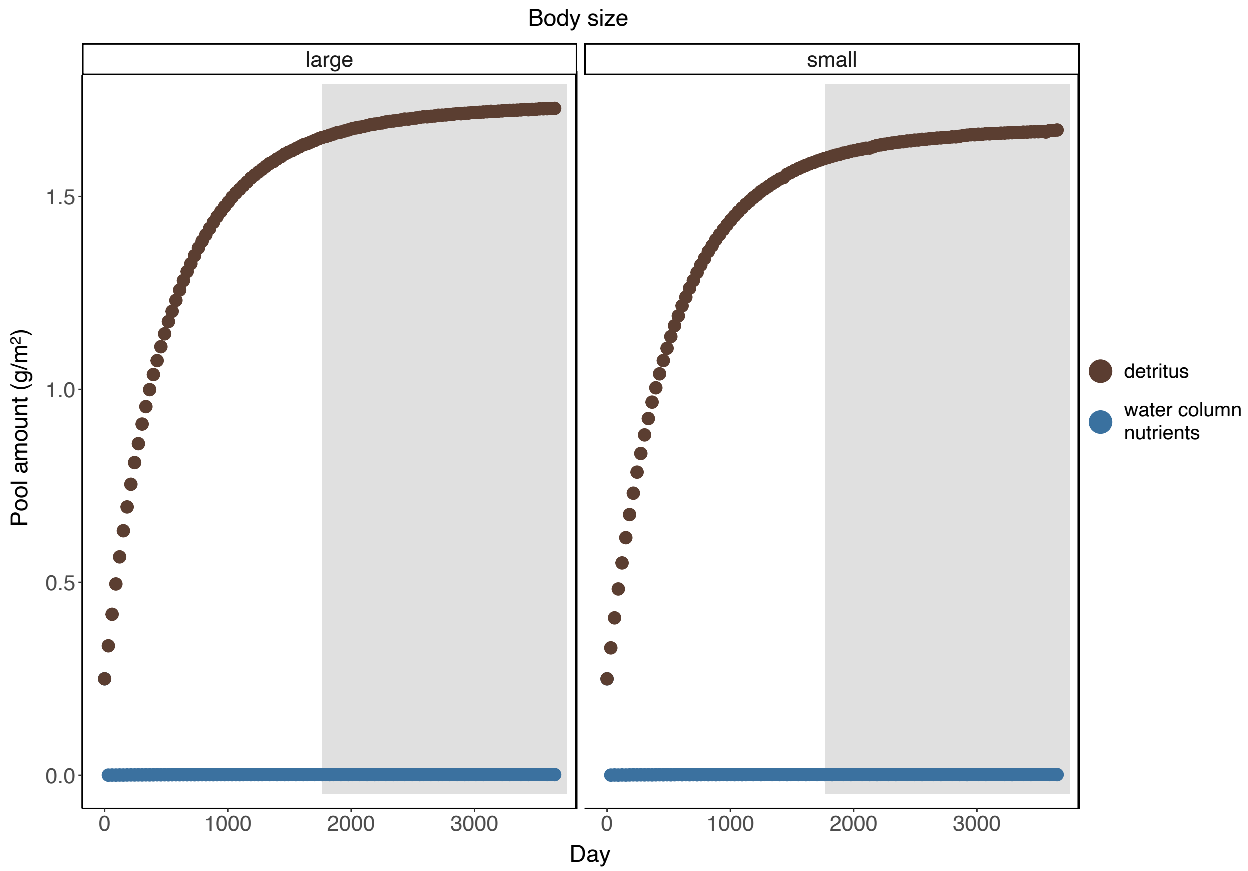

Figure S6. Detritus and water column nutrient pool amounts (g/m^2) for large- and small-bodied fish populations at medium biomass and medium foraging behavior. Pool amounts were calculated from all grid cells in the model environment for each timestep (~30 days) over a 10 year model (~3650 days). Each body size treatment was simulated 50 times and pool amounts were averaged across simulations. Colors represent pools (detritus and water column nutrients). The gray-shaded region signifies the final 5 years of the model run, the time period used for all analyses, to ensure the ecosystem had reached stable dynamics.

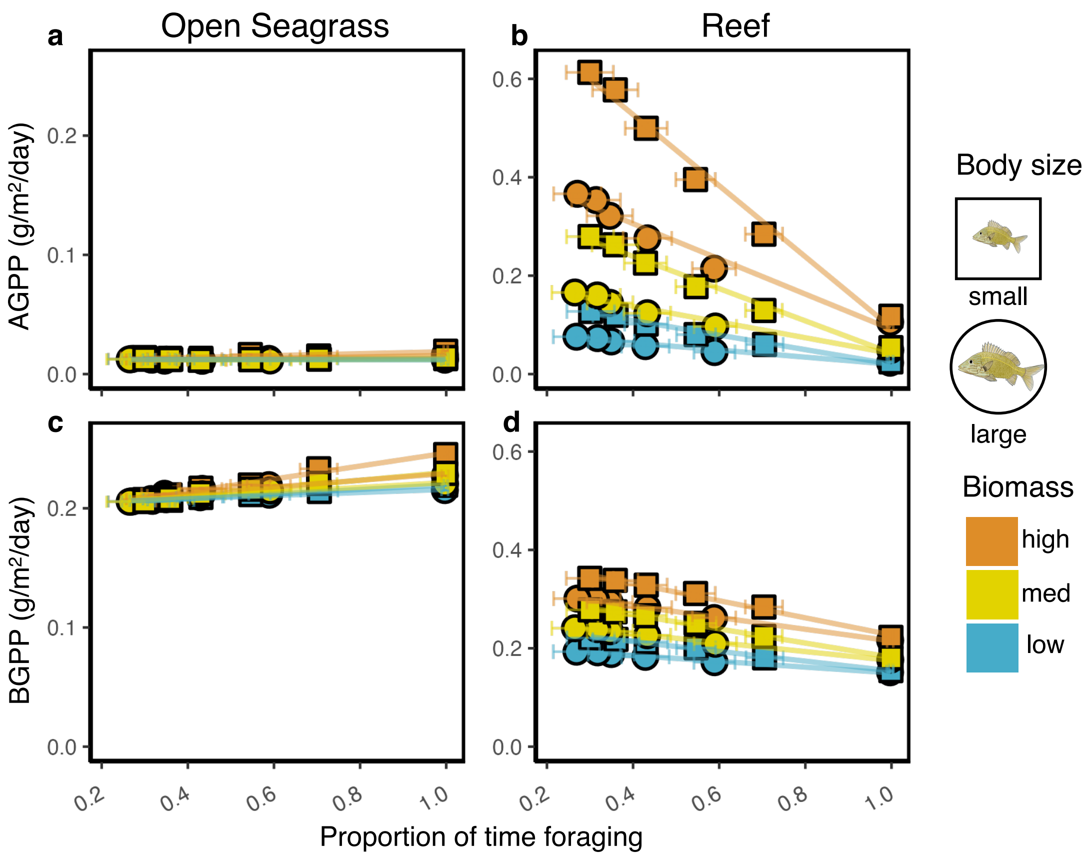

Figure S7. Effects of population-level foraging and body size on: (a) aboveground primary production (AGPP) in open seagrass (> 5 m from reef), (b) AGPP adjacent to the reef (< 5 m from reef), (c) belowground primary production (BGPP) in open seagrass, (d) BGPP adjacent to the reef. Values are means ± SD in production (y axis) and proportion of time individuals in a population spent foraging (x axis) across 50 iterations (note in both cases, SD is small and obscured by datapoints). Symbols represent body size (squares for small and circles for large) and colors represent biomass levels (low, medium, and high biomass). Note the different y-axes due to the spatial scale at which production was calculated: panels a and c were calculated from cells > 5 m from the reef (Open Seagrass) and panels b and d were calculated from cells < 5 m from the reef (Reef). Fish illustrations: Katrina S. Munsterman.

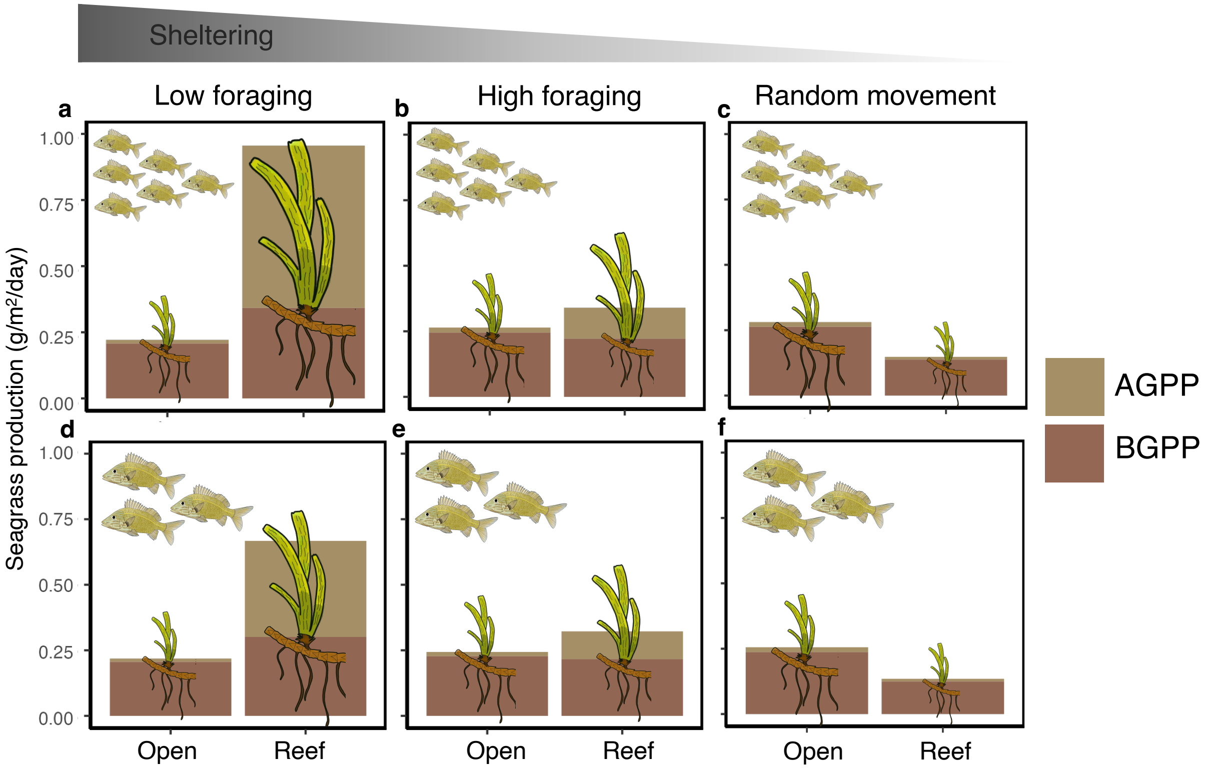

Figure S8. Proportion of seagrass production (g/m^2/day) from belowground primary production (BGPP) and aboveground primary production (AGPP) in open seagrass (> 5 m to reef) and seagrass adjacent to the reef (< 5 m to reef) in high biomass and small-bodied fish populations for (a) Low foraging, (b) High foraging, and (c) Random movement (i.e., not attracted to the reef to shelter), and high biomass and large-bodied fish populations for (d) Low foraging, (e) High foraging, and (f) Random movement. Fish and seagrass illustrations: Katrina S. Munsterman.

Table S1. Foraging behavior (population-level *Q1*, individual-level and population-level *Q2*), biomass, and body size parameters. Each parameter combination was simulated 50 times (1800 total simulations for *Q1*; 2700 total simulation for *Q2*).

Question 1 Population-level foraging behavior

**Pop-level foraging**		**Biomass**	**Body size**
Parameter *A*	Parameter *B*	Low	Small
0.99	0.0001	Medium	Large
0.25	0.01	High	
0.1	0.1		
0.05	0.5		
0.025	0.75		
0.01	0.99		

Question 2 Individual-level and population-level foraging behavior

**Ind-level foraging**	**Pop-level foraging**	**Biomass**	**Body size**
Bold	Low	Low	Small
Normal	Med	Medium	Large
Shy	High	High	

Table S2. **Q1 statistical model results.** Separate linear mixed-effects models were used to test how seagrass primary production (total, belowground, aboveground, reef-adjacent, and open seagrass) at high, medium, and low biomass was affected by body size, population-level foraging behavior, and their interaction. Partial eta-squared values were calculated to assess the proportion of variance explained by each predictor variable.

I. Total primary production

High biomass

	DF	Sum Sq	F-value	P value	Eta^2
**body size**	1	234.86	85842.92	< 0.001***	0.99
**pop-level foraging**	1	4.44	1622.23	< 0.001***	0.47
**size*foraging**	1	0.22	79.49	< 0.001***	0.12

Medium biomass

	DF	Sum Sq	F-value	P value	Eta^2
**body size**	1	62.65	225433.46	< 0.001***	1.00
**pop-level foraging**	1	5.14	18493.05	< 0.001***	0.93
**size*foraging**	1	0.04	158.15	< 0.001***	0.21

Low biomass

	DF	Sum Sq	F-value	P value	Eta^2
**body size**	1	81.92	2194.40	< 0.001***	0.78
**pop-level foraging**	1	439.43	11771.10	< 0.001***	0.83
**size*foraging**	1	53.13	1423.30	< 0.001***	0.70

II. Belowground primary production

High biomass

	DF	Sum Sq	F-value	P value	Eta^2
**body size**	1	81.92	2194.40	< 0.001***	0.78
**pop-level foraging**	1	429.43	11711.10	< 0.001***	0.83
**size*foraging**	1	53.13	1423.30	< 0.001***	0.70

Medium biomass

	DF	Sum Sq	F-value	P value	Eta^2
**body size**	1	32.69	3587.00	< 0.001***	0.86
**pop-level foraging**	1	169.04	18546.60	< 0.001***	0.91
**size*foraging**	1	10.77	1181.40	< 0.001***	0.66

Low biomass

	DF	Sum Sq	F-value	P value	Eta^2
**body size**	1	8.49	3475.08	< 0.001***	0.85
**pop-level foraging**	1	59.17	24201.95	< 0.001***	0.94
**size*foraging**	1	1.92	785.59	< 0.001***	0.57

III. Aboveground primary production

High biomass

	DF	Sum Sq	F-value	P value	Eta^2
**body size**	1	206.82	17114.00	< 0.001***	0.97
**pop-level foraging**	1	214.94	17787.00	< 0.001***	0.89
**size*foraging**	1	21.39	1770.00	< 0.001***	0.75

Medium biomass

	DF	Sum Sq	F-value	P value	Eta^2
**body size**	1	62.39	11872.20	< 0.001***	0.95
**pop-level foraging**	1	92.21	17545.60	< 0.001***	0.90
**size*foraging**	1	6.97	1325.70	< 0.001***	0.69

Low biomass

	DF	Sum Sq	F-value	P value	Eta^2
**body size**	1	16.26	9013.00	< 0.001***	0.94
**pop-level foraging**	1	28.06	1551.80	< 0.001***	0.90
**size*foraging**	1	1.47	816.30	< 0.001***	0.58

IV. Reef-adjacent (< 5 m) primary production

High biomass

	DF	Sum Sq	F-value	P value	Eta^2
**body size**	1	31.50	6306.10	< 0.001***	0.91
**pop-level foraging**	1	103.24	20665.2	< 0.001***	0.92
**size*foraging**	1	5.95	1190.90	< 0.001***	0.67

Medium biomass

	DF	Sum Sq	F-value	P value	Eta^2
**body size**	1	50.66	9070.70	< 0.001***	0.94
**pop-level foraging**	1	184.16	32971.70	< 0.001***	0.95
**size*foraging**	1	8.29	1484.70	< 0.001***	0.71

Low biomass

	DF	Sum Sq	F-value	P value	Eta^2
**body size**	1	69.87	11929.00	< 0.001***	0.95
**pop-level foraging**	1	269.70	46048.40	< 0.001***	0.96
**size*foraging**	1	9.94	1696.80	< 0.001***	0.74

V. Open seagrass (> 5 m) total primary production

High biomass

	DF	Sum Sq	F-value	P value	Eta^2
**body size**	1	79.86	996.68	< 0.001***	0.63
**pop-level foraging**	1	760.76	9494.29	< 0.001***	0.82
**size*foraging**	1	57.47	717.26	< 0.001***	0.55

Medium biomass

	DF	Sum Sq	F-value	P value	Eta^2
**body size**	1	18.03	626.12	< 0.001***	0.51
**pop-level foraging**	1	281.25	9767.72	< 0.001***	0.83
**size*foraging**	1	17.94	622.88	< 0.001***	0.51

Low biomass

	DF	Sum Sq	F-value	P value	Eta^2
**body size**	1	2.59	279.92	< 0.001***	0.31
**pop-level foraging**	1	99.95	10786.27	< 0.001***	0.85
**size*foraging**	1	5.77	622.66	< 0.001***	0.51

Table S3. Q2 statistical model results. Separate linear mixed-effects models were used to test how seagrass primary production (total, belowground, and aboveground) at high, medium, and low biomass was affected by body size, individual-level foraging behavior, population-level foraging behavior, and their interactions. Partial eta-squared values were calculated to assess the proportion of variance explained by each predictor variable.

I. Total primary production

High biomass

	DF	Sum Sq	F-value	P value	Eta^2
**body size**	1	414.79	3005016.10	< 0.001***	1.00
**ind-level foraging**	2	6.48	23463.20	< 0.001***	0.87
**pop-level foraging**	2	3.49	12631.60	< 0.001***	0.70
**size*ind*pop**	4	0.77	1387.10	< 0.001***	0.86

Medium biomass

	DF	Sum Sq	F-value	P value	Eta^2
**body size**	1	103.52	1105046.41	< 0.001***	0.99
**ind-level foraging**	2	6.38	34054.29	< 0.001***	0.96
**pop-level foraging**	2	3.98	21260.50	< 0.001***	0.94
**size*ind*pop**	4	0.07	177.02	< 0.001***	0.45

Low biomass

	DF	Sum Sq	F-value	P value	Eta^2
**body size**	1	23.39	213940.00	< 0.001***	0.96
**ind-level foraging**	2	4.48	20489.00	< 0.001***	0.95
**pop-level foraging**	2	2.92	13392.00	< 0.001***	0.94
**size*ind*pop**	4	0.003	6.86	< 0.001***	0.03

II. Belowground primary production

High biomass

	DF	Sum Sq	F-value	P value	Eta^2
**body size**	1	201.46	56286.10	< 0.001***	0.97
**ind-level foraging**	2	520.42	72599.90	< 0.001***	0.96
**pop-level foraging**	2	320.73	44805.20	< 0.001***	0.95
**size*ind*pop**	4	26.07	1820.90	< 0.001***	0.89

Medium biomass

	DF	Sum Sq	F-value	P value	Eta^2
**body size**	1	50.55	34041.39	< 0.001***	0.94
**ind-level foraging**	2	167.10	56263.27	< 0.001***	0.97
**pop-level foraging**	2	105.71	35592.31	< 0.001***	0.95
**size*ind*pop**	4	3.41	573.23	< 0.001***	0.72

Low biomass

	DF	Sum Sq	F-value	P value	Eta^2
**body size**	1	11.07	10760.65	< 0.001***	0.74
**ind-level foraging**	2	55.54	26999.87	< 0.001***	0.95
**pop-level foraging**	2	35.58	17295.91	< 0.001***	0.94
**size*ind*pop**	4	0.34	82.19	< 0.001***	0.27

III. Aboveground primary production

High biomass

	DF	Sum Sq	F-value	P value	Eta^2
**body size**	1	152.81	77851.60	< 0.001***	<0.01
**ind-level foraging**	2	360.84	91918.30	< 0.001***	0.98
**pop-level foraging**	2	233.76	59545.30	< 0.001***	0.97
**size*ind*pop**	4	12.61	1601.10	< 0.001***	0.88

Medium biomass

	DF	Sum Sq	F-value	P value	Eta^2
**body size**	1	57.16	60655.65	< 0.001***	0.28
**ind-level foraging**	2	126.73	67237.64	< 0.001***	0.97
**pop-level foraging**	2	82.79	43929.97	< 0.001***	0.96
**size*ind*pop**	4	2.59	686.99	< 0.001***	0.76

Low biomass

	DF	Sum Sq	F-value	P value	Eta^2
**body size**	1	17.52	28608.66	< 0.001***	0.58
**ind-level foraging**	2	37.09	30283.55	< 0.001***	0.95
**pop-level foraging**	2	23.86	19479.29	< 0.001***	0.94
**size*ind*pop**	4	0.35	141.37	< 0.001***	0.39
